# Repair of progressive retinal detachment complicating degenerative retinoschisis: surgical management and outcomes in phakic eyes

**DOI:** 10.1186/s40942-021-00344-2

**Published:** 2021-11-18

**Authors:** Bradley Beatson, Alex Pham, Sally S. Ong, Ishrat Ahmed, J. Fernando Arevalo, James T. Handa

**Affiliations:** Wilmer Eye Institute, Johns Hopkins School of Medicine, 400 N. Broadway; Smith Building 3015, Baltimore, MD 21287 USA

**Keywords:** Retinal detachment, Schisis detachment, Scleral buckle, Degenerative retinoschisis, Vitrectomy

## Abstract

**Background:**

Degenerative retinoschisis is a common condition defined by the splitting of the neurosensory retina that may rarely be associated with progressive retinal detachment (RD). Here, we aim to describe the anatomic and functional outcomes of surgical treatment of progressive symptomatic retinal detachment complicating degenerative retinoschisis (PSRDCR) using pars plana vitrectomy (PPV), scleral buckle (SB), or combined PPV/SB procedure.

**Methods:**

A retrospective chart review of patients with PSRDCR between Jan 1, 2008 and Dec 31, 2019 was conducted. Data regarding demographics, surgical approach, and anatomic/functional outcomes were collected.

**Results:**

Of the 4973 charts with RD repair during the study period, 36 eyes (0.7%) had retinoschisis with RD. 18 eyes met inclusion criteria (0.4%). The median age was 54 years (range 18–74) and all eyes were phakic. All eyes had outer layer breaks (OLBs) and 16 eyes (89%) had identifiable inner layer breaks. All OLBs were posterior to the equator in charts where position was recorded (16 eyes). The single surgery anatomic success (SSAS) and final anatomical success rates were 66% (12/18) and 100%, respectively. Eyes treated with PPV/SB had an SSAS rate of 75% (9/12), while PPV and SB had SSAS rates of 66% (2/3) and 33% (1/3), respectively.

**Conclusions:**

PSRDCR is an exceedingly rare complication of degenerative retinoschisis associated with an SSAS rate lower than for uncomplicated rhegmatogenous RD. The majority of PSRDCR were repaired via combined PPV/SB in our study, and the rarity of this complication limits statistical support of an optimal surgical method in our and prior studies. The role of SB combined with PPV for PSRDCR requires further investigation.

## Introduction

Degenerative (senile) retinoschisis, a cystic degeneration and splitting of the neurosensory retina in the outer plexiform layer, is common and estimated to affect approximately 4% of individuals aged 60–80 years [1]. In several natural history and case studies, retinoschisis has been shown to progress asymptomatically in the vast majority of cases and is thus conservatively managed [1–5]. Retinal detachment (RD) within the zone of schisis, which is most frequently located in the inferotemporal quadrant, is one complication of retinoschisis [3, 4]. These detachments develop due to the formation of an outer layer break (OLB) in the retina, allowing the fluid in the schisis cavity to enter the subretinal space [4]. Localized RDs, which occur in roughly 9% of patients with retinoschisis, usually do not progress outside of the schisis cavity and thus rarely require treatment [2]. With an incidence of 0.05% in patients with retinoschisis, an RD can progress beyond the borders of the schisis cavity with associated symptoms, which is designated as a progressive, symptomatic retinal detachment complicating retinoschisis (PSRDCR) [4]. These detachments are commonly associated with both OLBs and inner layer breaks (ILB), thereby allowing vitreous to enter the schisis cavity and subsequently the subretinal space [4]. In this scenario, immediate surgical intervention is indicated to preserve vision, especially when RD progression threatens the macula [4].

For PSRDCR associated with limited, peripheral OLBs anterior to the equator, scleral buckle (SB) with cryopexy has been shown to have a high success rate [2, 6–10]. However, for extensive PSRDCR with a large OLB or when extending posterior to the equator, a consensus on appropriate intervention has not been established. In 2013, a national survey of vitreoretinal surgeons in the United Kingdom revealed a significant variation in surgical intervention preference for different clinical scenarios involving PSRDCR [11]. Meanwhile, the literature comparing the efficacy of interventions for PSRDCR is sparse, and a number of case series have suggested using a variety of techniques, including pneumatic retinopexy, external drainage of the schisis cavity, barrier laser photocoagulation, cryotherapy, SB, and pars plana vitrectomy (PPV) [7, 9, 12–18]. Without definitive evidence, some authors have advocated PPV for extensive detachments because of the superior ability to visualize retinal breaks using the operating microscope [6, 8, 10, 13, 19]. Due to the rarity of PSRDCR, a prospective clinical trial to determine the best surgical approach is not feasible. Thus, the objective herein is to analyze the anatomic and functional outcomes of PSRDCR and retinal re-detachment in 18 phakic eyes (17 patients) that were repaired with SB, PPV, or a combined PPV/SB procedure.

## Materials and methods

A retrospective chart review of patients who underwent retinal detachment repair at the Wilmer Eye Institute from January 1, 2008, to December 31, 2019, was conducted. Those with primary repair of PSRDCR at Wilmer and a follow-up period of at least 6 months were included. Charts with diagnoses other than PSRDCR, such as juvenile X-linked retinoschisis or schisis-RD without progression, were excluded.

### Patient demographics and baseline characteristics

Preoperative data including age at presentation, gender, degree of myopia, refractive error, prior trauma, prior intraocular surgeries, lens status, posterior vitreous detachment (PVD) status, presence of pre-operative proliferative vitreoretinopathy (PVR), baseline best-corrected visual acuity (BCVA), and baseline intraocular pressure were collected.

### PSRDCR features

The following PSRDCR characteristics were recorded: extent and position of retinoschisis, foveal and macular involvement, extent and position of RD, and type, number, and location of retinal breaks, and the time to primary repair and total follow-up interval. Macula-off detachments were defined as detachments with mention of foveal involvement in patient charts.

### Surgical approaches

PSRDCRs were repaired using one of three surgical methods: combined PPV and SB, PPV alone, or SB alone. For primary and any subsequent repairs, the following were collected: type of surgical intervention, use of laser, cryopexy, and temporary perfluorocarbon (PFC) tamponade, drainage retinotomy, relaxing retinectomy, membrane peeling, and use of SF_6_, C_3_F_8_, silicone oil, or air tamponade. Either a #240 or #41 band were used for the buckle. When appropriate, the time to re-detachment, etiology of re-detachment, and the number of additional RD repairs required were recorded.

### Anatomic/functional outcomes and postoperative complications

The primary outcome is the single surgery anatomic success (SSAS) rate of each individual procedure used to repair PSRDCR. Single surgery anatomic success (SSAS) was defined as repaired PSRDCR and an attached retina that did not require additional reattachment surgery as of final follow-up. Final anatomic success was defined as an attached retina without presence of tamponade at final follow-up. From the time of primary repair to final follow up, other pertinent outcomes recorded include: anatomic attachment at final follow up, BCVA following primary repair at 1–2 months and final follow up within the study period, and post-operative complications. BCVA was expressed as Snellen ratios. Post-operative complications that were recorded included ocular hypotony/hypertension within 1 month of surgery, choroidal detachment, epiretinal membrane (ERM), cystoid macular edema (CME), diplopia or strabismus, macular atrophy, corneal decompensation, and cataract progression and surgery.

## Results

### Patient demographics and baseline characteristics

Of the 4973 charts reviewed with retinal detachment, 36 had retinoschisis with RD (0.7%). Eighteen eyes from 17 patients were included following the inclusion and exclusion criteria (0.4%). Case 11, who was 18 years old, had no macular schisis changes such as petaloid changes to suggest juvenile retinoschisis and thus was included. Nine different surgeons were involved in the primary RD repairs of these 18 eyes. Baseline clinical characteristics, characteristics of the primary detachment, and visual outcomes for each group are shown in Table 1.

**Table 1:** Patient baseline characteristics and visual outcomes grouped by primary surgery

	PPV/SB group	PPV group	SB group	Total
No. of eyes	12	3	3	18
No. of patients	11	3	3	17
Mean age, years	51.3	60.3	42.7	51.3
Sex, n (%)				
Male	4 (36.4)	2 (66.6)	1 (33.3)	7 (41.2)
Female	7 (63.6)	1 (33.3)	2 (66.6)	10 (58.8)
Median BCVA (mean)				
Baseline	20/32 (20/44)	20/32 (20/36)	20/20 (20/43)	20/32 (20/46)
Final	20/40 (20/58)	20/25 (20/39)	20/20 (20/147)	20/32 (20/64)
Median extent of detachment, clock hours (mean)	5.75 (5.0)	4 (4.5)	2.5 (2)	4.4
Macula-involving detachments, n (%)	6 (50)	1 (33)	1 (33)	8 (44)
Posterior vitreous detachment, n (%)	7 (58)	2 (66)	0 (0)	9 (50)
Tamponade, n (%)				
Sterile air or none	1 (8.3)	0	3 (100)	3 (16.7)
SF_6_	7 (58.3)	3 (100)	0	10 (66.6)
C_3_F_8_	4 (33.3)	0	0	5 (27.8)
Median follow-up, years (mean)	5.0 (5.0)	2.3 (4.2)	2.8 (4.2)	2.8 (4.8)
Eyes redetached (%)	3 (25%)	1 (33%)	2 (66%)	6 (33%)

*PPV *pars plana vitrectomy; *SB *scleral buckle; *BCVA *best-corrected visual acuity

Individual patient clinical data is presented in Table 2. Patients ranged in age from 18 to 74 years (median, 54 years). At the time of preoperative presentation, no eye had a prior trauma. No eye had a history of prior intraocular surgery except for case 6, which underwent PPV for symptomatic, progressive bullous retinoschisis without retinal detachment. The retina detached 17 days later, at which point the primary RD repair was performed for PSRDCR. Thirteen eyes had a history of myopia, with a median of − 4.5 diopters (range − 3 to − 6.75). At initial presentation, all patients were phakic, 10 patients presented with cataract (6 patients with 1  +  nuclear sclerosis, 4 patients with 2  +  nuclear sclerosis), and no patients had PVR of at least grade B. Two patients, cases 1 and 13, had preoperative PVR grade A.

**Table 2:** Patient clinical data

Case	Age (y)/sex	Follow-up (mo)	Primary surgery	Tamponade used	Primary reattachment at final follow-up	Position of primary detachment	No. of OLBs	Position of OLBs	No. of ILBs	Position of ILBs
1^a^	53/F	115	PPV/SB	SF6	Yes	IT	4	T	Multiple	T
2^a^	54/F	115	PPV/SB	SF6	No	IT	Multiple	T	Multiple	T
3	44/F	65	PPV/SB	Sterile air	Yes	IT	Multiple	IT	5	IT
4	35/M	32	PPV/SB	C3F8	Yes	I	1	I	1	T
5	56/F	18	PPV/SB	C3F8	Yes	T	> 4	S	1	ST
6	69/M	19	PPV/SB	C3F8	No	I	1	I	> 4	I
7	64/F	108	PPV/SB	SF6	Yes	ST	1	T	0	
8	54/F	19	PPV/SB	C3F8	No	I	1	I	5	I
9^b^	58/M	55	PPV/SB	SF6	Yes	ST	2	ST	1	ST
10	52/F	23	PPV/SB	SF6	Yes	I	1	I	Multiple	I
11	18/M	75	PPV/SB	SF6	Yes	I	1	I	4	T
12	58/F	76	PPV/SB	SF6	Yes	I	Multiple	I	3	I
13	74/M	28	PPV	SF6	Yes	T	1	ST	1	ST
14	48/M	25	PPV	SF6	No	I	1	IT	1	I
15	59/F	98	PPV	SF6	Yes	ST	1	S	2	S
16	30/F	33	SB	None	Yes	ST	1	S	Multiple	ST
17^b^	72/M	109	SB	None	No	I	1	T	2	T
18	26/F	8	SB	Sterile air	No	T	2	T	0	

Multiple =   ≥ 2, but exact number not specified in patient chart

*F * female; *M *male; *PPV *pars plana vitrectomy; *SB *scleral buckle; *OLB *outer layer break; *ILB *inner layer break; *T *temporal; *I *inferior; *S *superior; *N *nasal; *IT *inferotemporal; *ST *superotemporal

^a^Cases 1 and 2 were fellow eyes from the same patient

^b^Case 17 additionally had a single temporal horseshoe tear, while case 9 had a single superior horseshoe tear

### PSRDCR features

The preoperative duration of retinoschisis was unknown for most eyes. Seven cases (39%) had retinoschisis diagnosed when presenting urgently for PSRDCR, and 5 cases (28%) had retinoschisis of unknown duration prior to presenting with PSRDCR. Of the 6 eyes (33%) with known duration of retinoschisis before primary RD repair, the median interval between retinoschisis diagnosis to primary RD repair was 9.5 months (range 4–67 months). Of the 18 eyes that developed PSRDCR, 10 (56%) detachments were temporally located (with 3 inferotemporal and 4 superotemporal) and 8 (44%) were inferiorly positioned. All eyes had at least one OLB and 7 eyes (39%) had multiple (≥  2) OLBs. The location of OLBs was recorded for 16 eyes (89%), and at least one OLB was posterior to the equator in all of these eyes. For two of the eyes, the location of the OLBs was not specifically specified to determine if they were anterior or posterior to the equator. Both of these eyes were in the PPV/SB treatment group. Sixteen eyes (89%) had at least one ILB and 11 (64%) eyes had multiple (≥  2) ILBs. Five eyes (28%) had lattice degeneration without an associated retinal tear.

### Surgical approaches

The SSAS rates for PPV/SB, PPV, and SB were 9/12 (75%), 2/3 (66%), and 1/3 (33%), respectively. Although most of the cases had a preexisting cataract (10 of 18 eyes), none underwent combined phacoemulsification or lens extraction with primary repair. The method of subretinal fluid drainage to reattach the retina in the PPV/SB group was by internal drainage either through a pre-existing break (n  = 11 cases) or after creating a drainage retinotomy during application of PFO (n  = 11 cases) or during fluid-air exchange (cases 4 and 6). In the PPV group, subretinal fluid was internally drained through a pre-existing break during fluid-air exchange in all three cases, and a drainage retinotomy in one case (case 14). All three SB cases were performed without draining subretinal fluid. SF_6_ tamponade was used in 10/18 cases, C_3_F_8_ in 5/18 cases, and sterile air or none in 3/18 cases. The mean  ±  standard deviation (SD) time interval from first presentation of PSRDCR until primary repair was 9.7 (± 23.3) days.

### Anatomic outcomes and complications

Of the 18 PSRDCR cases, the mean (±  SD) follow-up after primary surgical repair was 57 (± 39) months, ranging from 8 to 115 months. At the final follow-up visit, all eyes were attached, representing a final anatomical success rate of 100%. The overall SSAS rate was 12/18 (66%). The 6 eyes that redetached underwent subsequent vitreoretinal surgery, as described in Table 3.

**Table 3:** Repair for patients with redetachment

Case	Time until first re-detachment (days)	Primary surgery (tamponade)	Reason for first re-detachment	Secondary surgery (tamponade)	Location of secondary RD	Total # RD surgeries
2	7	PPV/SB (SF_6_)	New tear	PPV/SB (SF_6_)	IT	2
6	36	PPV/SB (C_3_F_8_)	PVR	PPV (C_3_F_8_)	ST	3
8	57	PPV/SB (C_3_F_8_)	New tear	PPV (SF_6_)	S	2
14	46	PPV (SF_6_)	Lifted old tear	PPV (silicone oil)	T	2
17	23	SB (N/A)	SRF in schisis cavity, no tear noted	PPV (SF_6_)	IT	5
18	14	SB (sterile air)	New tear	SB, radial element (N/A)	IT	2

*PPV* pars plana vitrectomy; *SB *scleral buckle; *PVR *proliferative vitreous retinopathy; *SRF *subretinal fluid; *IT *inferotemporal; *ST *superotemporal; *S *superior; *RD *retinal detachment

Of these initial redetachments, 2/6 (33%), were macula-off. Both cases, cases 14 and 17, presented initially with macula-off primary detachments. New retinal tears were responsible for 3/6 of the initial redetachments. In case 2, the new tear was located inferiorly at the vitreous base. In case 8, the new tear was located superiorly, in the periphery on the anterior margin of the SB. The new tear in case 18 was located inferiorly. The anterior or posterior location of this tear was not noted. The total number of RD repair surgeries at final follow-up ranged from 2 to 5 (median, 2). Overall, the mean (±  SD) time to re-detachment was 30.5 (± 19.3) days. Eyes that had C_3_F_8_ tamponade in the primary repair detached later [mean (±  SD) = 46.5 (± 14.8) days] than eyes that had SF_6_ tamponade [mean (±  SD]  =  26.5 (±  27.6) days]. Two eyes (cases 6 and 17) required more than two RD repairs. Case 6 required 3 repairs, while case 17 required 5 repairs. For the third RD repair, case 6 developed PVR and underwent PPV with membrane peeling and relaxing retinotomy. Silicone oil tamponade was placed, which was successfully removed 7 months later. The third RD repair for case 17 was performed with PPV, the fourth via combined PPV/lensectomy/peripheral iridectomy/membrane peel/silicone oil, and the final RD repair was via PPV with aspiration of subretinal PFO and placement of silicone oil. No eyes had silicone oil in place by time of final-follow up.

### Complications

There were no intraoperative complications in any of the primary RD repairs. However, one intraoperative complication was identified in a subsequent RD repair. In Case 17, surgery 4, PFO was retained, which was seen postoperatively in the anterior chamber and later in the subretinal space after development of a recurrent RD with PVR. The PFO was removed during the fifth surgery to repair the RD/PVR. Postoperative complications for all eyes are shown in Table 4. The most common complication was cataract. Out of those without a preexisting cataract, 75% (6/8) developed a cataract after primary surgical repair. The remaining two eyes that did not develop a cataract were initially repaired with SB. Fourteen eyes (78%) had cataract surgery following primary RD repair and the mean (SD) time to surgery was 11 [12] months, ranging from 2 to 42 months. Of the eyes that did not redetach after the primary RD repair, 7/12 eyes (58%) developed postoperative complications. Conversely, 5/6 cases (83%) with re-detachment had postoperative complications.

**Table 4:** Patient functional outcomes

Case	Macula state	Baseline BCVA	3–7 mo postop BCVA	Final BCVA	Post-op complications, additional procedures	Cataract grade before surgery	Cataract formation after surgery	Time from primary RD repair to cataract surgery (mo)
1	Off	20/32	20/100	20/25	N/A	1 + NS		9
2	On	20/16	20/80	20/30	ERM; MA	1 + NS		3
3	On	20/15	20/20	20/30	AACG; SB extrusion and removal	N/A	Yes	N/A
4	Off	20/50	N/A	20/60	CME	N/A	Yes	31
5	Off	20/250	20/100	20/100	CME, ERM	N/A	Yes	N/A
6	Off	20/40	HM	20/200	PVR, ERM, CME, lensectomy, relaxing retinectomy	2 + NS		2
7	On	N/A	20/25	20/25	ERM	1 + NS		3
8	On	20/20	20/125	20/20	CME	N/A	Yes	17
9	On	20/20	20/200	20/125	CME, ERM	N/A	Yes	6
10	Off	20/50	N/A	20/200	ERM	2 + NS		Unknown
11	Off	20/63	20/160	20/50	N/A	N/A	Yes	9
12	On	20/32	20/40	20/20	N/A	1 + NS		42
13	On	20/30	20/40	20/16	N/A	1 + NS		7
14	Off	20/80	20/200	20/150	ERM, drainage retinotomy	1 + NS		7
15	On	20/20	20/200	20/25	N/A	2 + NS		7
16	On	20/20	N/A	20/20	N/A	N/A	No	N/A
17	Off	20/250	CF at 6′	HM	PVR, ERM, MA, lensectomy, anterior chamber silicone oil, corneal edema	2 + NS		5
18	On	20/16	20/25	20/20	N/A	N/A	No	N/A

*N/A *not available; *BCVA *best-corrected visual acuity; *NS *nuclear sclerosis; *RD *retinal detachment; *CF * count fingers; *HM *hand motion; *ERM *epiretinal membrane; *MA *macular atrophy; *AACG *acute angle closure glaucoma; *CME *cystoid macular edema; *PVR *proliferative vitreoretinopathy; *SRF *subretinal fluid

### Functional outcomes

BCVA at baseline, post-operative month 1, and final-follow up for each case is shown in Table 4. The median BCVA for macula-on detachments (n  = 10) was 20/20 at baseline and 20/25 at final follow-up. Macula-off detachments (n  = 8) had a median BCVA of 20/50 at baseline and 20/100 at final follow-up.

## Discussion

The treatment of PSRDCR is controversial in part due to its rare occurrence, with an estimated annual incidence of 0.85 persons per million [16]. Consequentially, no surgical method has emerged as superior for the treatment of PSRDCR due to the low sample size and statistical power in previous studies [6, 8, 16–18]. Of the 4973 charts with RD repairs from Jan 1, 2008 to Dec 31, 2019, only 18 (0.4%) met inclusion criteria for this study. In our cohort, eyes with macula-on detachments fared better, with a baseline median BCVA of 20/20 and final BCVA of 20/25. Eyes with macula-off detachments conversely presented with a median baseline BCVA of 20/50 and obtained a final BCVA of 20/100. As with primary RRD, not surprisingly, our results suggest that even following repair, macular involvement is associated with notably worse outcomes. While eyes in this case series had a 100% final reattachment rate, the SSAS rate was 66%, which is significantly lower than for the repair of uncomplicated rhegmatogenous retinal detachment (RRD) [20]. Similar success rates, ranging from 62 to 86%, have been seen for other recent PSRDCR case series, demonstrating that PSRDCR repair is associated with comparatively poor outcomes [6, 8, 10, 16–19]. While multiple retrospective studies have attempted to identify an optimal surgical procedure and have looked at the poor visual outcomes associated with PSRDCR, the discussion has been limited regarding the prognostic factors that may be associated with the low SSAS rates [6, 8, 16, 17]. Thus, in addition to discussing the characteristics of our cohort, we propose hypothetical factors that may influence the outcomes, such as preoperative lens status and the role of vitreous base traction.

Recent studies have advocated for SB or PPV to repair PSRDCR [6, 7, 10, 13, 16, 19, 21]. It is generally accepted that SB can be used in PSRDCR with anterior/peripheral OLBs or less extensive detachments with a high success rate [2, 6, 8, 21]. Conversely, PPV is often recommended for PSRDCR with posterior OLBs or extensive detachments, largely due to the improved visualization of the retina using the operative microscope. A combined PPV/SB procedure has also been utilized. Many studies have compared the outcomes of these procedures in order to identify the optimal surgical method, although current surgeon preference for PPV  ±  SB in more extensive PSRDCR introduces selection bias in retrospective studies attempting to compare surgical outcomes [6, 8, 10, 11, 16, 19, 21]. This bias was likely evident in our case series, as 50% of RDs treated with combined PPV/SB were macula-involving, while 33% of RDs treated with either PPV or SB were macula-involving. Similarly, patients with a combined primary procedure had a median detachment extent of 5.75 clock-hours, while those with PPV and SB alone had a median extent of 4 and 2.5 clock-hours, respectively. While the procedure with the highest SSAS rate in our study was combined PPV/SB, the low number of eyes treated with primary SB or PPV alone prevents any statistical conclusions regarding the most effective method of treatment.

Our cohort was comprised solely of phakic patients. In several previous studies on PSRDCR, the preoperative lens status was not reported, although some did have cohorts composed of mostly phakic patients [6, 8, 16, 17, 19, 21]. In the studies that identified pseudophakic patients, the outcomes based on lens status were not reported [10, 17]. We propose that the phakic lens status and cataract in this cohort may have influenced the surgical outcome. Since over half of the eyes in our study had cataract, albeit not severe enough to warrant lensectomy or concurrent cataract surgery, it is theoretically possible that the lens opacity impaired the surgeon’s ability to identify small retinal breaks or ILBs, especially given the small size and multitude of ILBs in PSRDCR. While no data are available on the SSAS rate with phacoemulsification during the repair of PSRDCR, the high rate of phakic patients raises the question of whether combined PPV/phacoemulsification will improve the SSAS rate. While care must be taken when extrapolating to a different disease, we look towards analogous data for uncomplicated RRD to suggest a benefit of this approach. Specifically, Caiado et al. [22] found a higher reoperation rate in phakic eyes treated with PPV for RRD relative to phakic eyes undergoing PPV/phacoemulsification. Smith et al. [23] and Caiado et al. [22] suggested that combined PPV/phacoemulsification/lens extraction may improve visualization of the retina in patients even without significant preexisting lens opacity. Further studies are needed to demonstrate the relevance of these findings to PSRDCR, as this combined procedure has yet to be systematically studied for PSRDCR.

New retinal tears caused half (3 of 6 eyes) of the redetachments. We speculate that the incomplete removal of peripheral vitreous in these phakic eyes led to vitreous base contraction that induced traction sufficient to either cause a new retinal tear or the progression of unrecognized or inadequately treated breaks during retinal re-detachment. Interestingly, Baron and Randriamora, who studied 52 eyes with retinoschisis, found that vitreous traction contributes to the development of ILBs [24]. Previously, PVD was proposed to have a central role in the pathogenesis of PSRDCR [16]. However, only 50% of our cases presented with PVD. While PVD may not have been present, we suggest that PSRDCR can develop when liquefied vitreous enters the subretinal space through both the inner and outer layer defects. While vitreous traction could contribute, a RRD in retinoschisis can occur in the absence of a PVD. Importantly, lens removal would enable careful vitreous base shaving that would minimize vitreous base contraction and the generation of new retinal tears or ILBs. Vitreous base shaving may be comparatively limited in phakic eyes, even with scleral indentation. While the use of SB alone in extensive PSRDCR is controversial, conceptually, the addition of SB with PPV to reduce peripheral vitreous traction offers an alternative approach to lens removal. In our series, 17 of 18 eyes eventually had a SB placed, which could have contributed to the 100% final reattachment rate. Thus, while SB alone as a procedure of choice is unresolved, our data suggest that it could be beneficial when combined with PPV.

The strengths of this study include the multiple surgeons that may simulate “real life circumstances” and the long follow-up (mean of 4.8 years), with most patients having over 2 years of follow-up. This period represents a follow-up time longer than other published PSRDCR case series. This study is limited by the small sample size that prevented us from making any statistical conclusions regarding the relative effectiveness of surgical procedures, which is a flaw of currently published PSRDCR studies [6, 8, 10, 13–19, 21]. Due to the inability to draw statistical conclusion regarding optimal surgical method, the goal of this work is to suggest future direction for investigation in the treatment of PSRDCR given the data obtained, with the ultimate goal of identifying a surgical method that will help mitigate the exceeding low SSAS rate for PSRDCR seen in recently published studies [6, 8, 10, 16–19].

## Conclusions

Eighteen cases (0.4%) of PSRDCR were identified in 4973 patient charts with RD repair over a 12-year period. The majority of PSRDCR in our cohort were repaired using combined PPV/SB, although the sample size was not large enough to compare the efficacy of alternative surgical methods. The low SSAS rate associated with this complication reaffirms the need for further research and discussion in order to identify prognostic factors linked to poor visual and anatomic outcomes.

## Data Availability

Not applicable.

## Abbreviations

PSRDCR: Progressive symptomatic retinal detachment complicating degenerative retinoschisis
PPV: Pars plana vitrectomy
SB: Scleral buckle
RD: Retinal detachment
OLB: Outer layer break
SSAS: Single surgery anatomical success
